# Why is spirometry underused in the diagnosis of the breathless patient: a qualitative study

**DOI:** 10.1186/1471-2466-11-37

**Published:** 2011-06-16

**Authors:** Nicola J Roberts, Susan F Smith, Martyn R Partridge

**Affiliations:** 1 Health Economics and Health Technology Assessment, Centre for Population & Health Sciences, University of Glasgow, 1 Lilybank Gardens, Glasgow, G12 8RZ, UK; 2 Imperial College London, Guy Scadding Building, Royal Brompton Campus, Dovehouse Street, London, SW3 6LY, UK; 3 Faculty Education Office (Medicine), Imperial College London, Sir Alexander Fleming Building, South Kensington Campus, London, SW7 2AZ, UK

**Keywords:** Spirometry, Trainees, General Practitioners, Barriers to use

## Abstract

**Background:**

Use of spirometry is essential for the accurate diagnosis of respiratory disease but it is underused in both primary and specialist care. In the current study, we have explored the reasons for this underuse.

**Methods:**

Five separate focus groups were undertaken with final year medical undergraduates, junior hospital doctors, general practitioners (GPs) and specialist trainees in respiratory medicine. The participants were not told prior to the session that we were specifically interested in their views about spirometry but discussion was moderated to elicit their approaches to the diagnosis of a breathless patient, their use of investigations and their learning preferences.

**Results:**

Undergraduates and junior doctors rarely had a systematic approach towards the breathless patient and tended, unless prompted, to focus on the emergency room situation rather than on patients with longer term causes of breathlessness. Whilst their theoretical knowledge embraced the possibility of a non-respiratory cause for breathlessness, neither undergraduates nor junior doctors spontaneously mentioned the use of spirometry in the diagnosis of respiratory disease. When prompted they cited lack of familiarity with the use and location of equipment, and lack of encouragement to use it as being major barriers to utilization. In contrast, GPs and specialist respiratory trainees were enthusiastic about its use and perceived spirometry as a core element of the diagnostic workup.

**Conclusions:**

More explicit training is needed regarding the role of spirometry in the diagnosis and management of those with lung disease and this necessitates both practical experience and training in interpretation of the data. However, formal teaching is likely to be undermined in practice, if the concept is not strongly promoted by the senior staff who act as role models and trainers.

## Background

There are over 40 common respiratory conditions many of which share symptoms with disorders of other systems. Breathlessness for example may be due to heart or lung disease, diaphragm weakness, pulmonary vascular disease or systemic disorders such as anaemia, obesity or hyperthyroidism. The correct differentiation requires a systematic approach which may develop with experience but ideally should be taught to trainees. Accurate diagnosis often includes the appropriate use of relevant investigations. Failure to harness one powerful investigative tool, spirometry, may lead to both misdiagnosis and under diagnosis of common conditions such as chronic obstructive pulmonary disease (COPD) [1]. Despite the importance of spirometry, studies from a number of countries indicate that it is frequently underused in both hospital and primary care settings [2-5].

One Belgian Study of patients with presumed obstructive lung disease being managed in primary care found that only one third had undergone spirometry in the previous two years [6]. Similarly, a study of 25 GP practices in the USA found that 75% failed to use spirometry in their diagnosis of COPD [7], despite other observations that diagnoses made in the absence of spirometry are frequently flawed [1]. The reasons cited for non-use include lack of time and staffing [7]. A longitudinal study in Denmark demonstrated that improved education of staff enhanced the use of spirometry in hospital outpatients with COPD, indicating the importance of staff training [8].

Availability of equipment in primary care seems to be a less important factor, with spirometers often being available, but not used. A study of Australian general practitioners (GPs) found that whilst almost 75% reported having a spirometer in their practice, only 12% had used it to review the majority of their patients with asthma within the year prior to the study [9]. Similar underuse has been observed in primary care in Sweden [10] and in Spain, where, although 50 of 55 primary care centres investigated possessed a spirometer, 11 never used it and only 2 performed more than 10 tests per week [11]. A recent study produced more optimistic conclusions with the authors reporting that 74% of primary care physicians responding to a questionnaire said that they used spirometry in the diagnosis of COPD [12] although the actual frequency of use was not measured.

Underuse is not limited to primary care. A study in Johns Hopkins Hospital concluded that airway obstruction was seriously under-diagnosed in hospitalised patients, not only at the time of admission, but that it remained undiagnosed and therefore untreated, at the time of discharge [4]. The authors concluded that routine use of spirometry would reduce this problem [4]. A study of patients with cardiovascular disorders in Italy reached a similar conclusion [13].

Thus, despite the wealth of evidence supporting the value of spirometry as a diagnostic and staging tool, and the enthusiasm with which its use is promoted in guidelines [14] there is a clear disconnect between recommendation and practice. Factors suggested to explain this include lack of time and inadequate staff training [7]. Any professional intending to use spirometry should be trained in both performance of the test and in interpreting the findings [15].

Published evidence indicates that in addition to spirometry being underused, its interpretation is often poorly understood by junior doctors [4,16] and a lack of confidence may thus contribute to under use. Another possibility may be that not all potential users accept the value of spirometry as a tool which will impact on practice or patient welfare [17] and, since COPD is largely a condition of smokers, it has been reported that some doctors fail to use spirometry since they believe that little or nothing can be done to help patients who continue to smoke [18]. A previous study in 2005 [19], reported barriers to the use of spirometry to include poorly designed and unduly complex spirometers which offer too many confusing parameters of limited value, lack of availability of spirometers, poor or no teaching in medical schools and the perceived lack of an evidence base demonstrating the value and cost-effectiveness of spirometry.

The aim of the current study was to investigate one of these potential barriers to the appropriate use of spirometry in the diagnosis of the breathless patient, that of physician education. In order to do this, a series of focus groups were conducted with three groups of medical professionals at early stages of their training to investigate what teaching on spirometry they recalled receiving and the extent to which they used it. Two further groups were conducted with more senior physicians who were either training as respiratory specialists or were established primary care physicians.

## Methods

An independent facilitator ran 5 separate one hour focus group sessions. Three groups consisted of non-specialised trainees [final year medical undergraduates (n = 6, UGs), junior doctors (n = 8, 5 pre registration trainees {Foundation Year 1} [F1s] and 3 senior house officers {Foundation Year 2}[F2s])], whilst the remaining two were with general practitioners with a special interest in undergraduate education (n = 8, GPs) and specialist registrars in respiratory medicine (n = 6, SpRs). Each group was drawn from a single category of professionals partly to optimize open discussion and minimize inhibition arising from formation of internal hierarchies, but also in the hope that each group would bring its own perspective to bear on the questions posed. Undergraduate participants were recruited by placing an advertisement on the Imperial College Medical Student Union website and were all Imperial students. F1s, F2s and SpRs were recruited during weekly trainee teaching sessions and were all drawn from the same London based NHS Trust, whilst the GPs were recruited by personal invitation from a group attending Imperial College to be updated on the undergraduate teaching programme. The same facilitator, a research nurse not otherwise involved in the study, moderated all groups and elicited participants' views on each of the following topics, discussed in the order listed below:

• General approach to the diagnosis of the breathless patient

• Classification of the causes of breathlessness

• Methods and investigations used as aids to diagnosis of the breathless patient

• Value and accessibility of spirometry, and of its interpretation

• How individuals learnt best about respiratory medicine

None of the participants were told prior to the session that we were specifically interested in their views about spirometry. Sessions were recorded and transcribed, then themed by all three authors independently using published methods [20]. Each author had an unmarked copy of each transcript and working independently, thematically coded the transcripts manually, following repeated private readings. Authors did not necessarily code transcripts in the same sequence. Following coding the authors compared notes and held extensive discussions about the themes identified. The same themes occurred in all focus groups and were independently identified by all authors.

### Ethical approval

This study underwent ethical review and permission was granted by the Head of Undergraduate Education, Faculty of Medicine, Imperial College London, and the Head of the North West Thames Foundation School, in accordance with the formal procedure in place for review of educational studies at this institution at the time the work was undertaken in 2004/5. It was deemed by them to be primarily an evaluation of teaching methods. The Imperial College Research Ethics Committee for the review of studies involving human subjects who were not patients, was not created until 2006.

## Results

It was striking that, when asked specifically about the methods and investigations used as aids to the diagnosis of breathlessness, none of the junior trainees mentioned spirometry spontaneously, whilst specialist registrars and GPs perceived spirometry as a fundamental element of their diagnostic work-up. The main factors inhibiting undergraduates and junior postgraduate trainees from using spirometry included lack of familiarity with equipment (17 comments), lack of encouragement from senior colleagues (6 comments) and lack of access to equipment (5 comments).

### General approach to the breathless patient

When asked about their general approach to the breathless patient, medical students focused on severe, acute admissions in the emergency room, obviously utilising an algorithmic approach, but one that focused on resuscitation not on diagnosis.

"It's alright if they are completely unconscious because you just go straight down the A B C line [Airway, Breathing, Circulation] and you know, you forget about taking a history, you get on to doing the resuscitation type thing," (UG-4)

They reported a lack of self-confidence in their ability to manage a breathless patient optimally in this setting.

"But it's knowing as a medical student, what the key questions to ask and knowing when to stop taking the history and get on with the management and it is having the confidence to say "right I will come back later and find out more about you" for clerking and let's go straight in there and do something" (UG-4 )

Even when asked to specifically to consider the non-acute case, juniors did not mention spirometry. In contrast, F1, F2 and SpRs focused on the chronically breathless patient, but commented that differential diagnosis in clinical practice was harder than the cases presented at medical school.

"...everything we do in medical school prepares you for it being much more easy to distinguish, rather... you know, whereas it's not all that easy..." (F2-2)

### Classification of breathlessness

When classifying causes of breathlessness, all groups (28 subjects) started by differentiating between urgent and non-urgent (14 comments) and (encouragingly) all mentioned the possibility of non-respiratory causes for breathlessness (16 comments).

"Well, I have just... very broadly the first thing that comes to mind, I mean, is does that patient have any respiratory disease or cardiac disease, are they anaemic or is it functional?" (SPR-1)

Worryingly, no group reported an overt strategy for arriving at a diagnosis and junior trainees tended to rely on their knowledge of what was most common to deduce what was most probable. These groups also had a tendency when in the emergency room to rely on prior observations and investigations made by ambulance paramedics at the time of admission.

"When the patient comes through the door you are told by the ambulance driver or the paramedic "this patient has a normal blood sugar", "this is their ECG it looks normal to me" and they automatically kind of lead you down the right path because they have told you a couple of things that it is not, so you can just get on with asking other questions." (UG-4)

### Use of spirometry

Only SpRs and GPs with a special interest in undergraduate education spontaneously cited spirometry as a diagnostic tool.

"Spirometry, I think lung function for me is always so, it is so important" (SPR-1)

"I use it so much that you almost forget that it's a you know, a thing that you have to think about doing, because you'd never see a new patient without spirometry" SPR-2:

When asked explicitly about spirometry, other grades cited unfamiliarity and inability to interpret the results as key factors inhibiting their use of spirometry (26 comments from 14 people).

F2s specifically noted lack of encouragement, reinforcement, or even basic information about obtaining spirometric equipment from senior colleagues, whereas respiratory SpRs and GPs viewed spirometry as essential.

"I have stopped doing it [spirometry] because we never got any sort of feedback". (F2-1)

If, during their training, undergraduates are taught about spirometry and perform lung function measurements using equipment of a type unlikely to be found on wards or in GP surgeries, they may well have difficulty working out how to use it, or even identifying it, later on in their training.

"The things that stop you is actually finding it in the department.. you know, finding someone who can help you use it.. because I had never used one before of that type..."(UG-3)

"I remember on my last shadow, the registrar told the house office to get spirometry done, some lung function tests done and it was just the house officer even felt in a bit of a tizz, didn't know where to order the tests from.... How would you?. It was just a different world and I have to say I would probably kind of feel the same. " (UG-5)

GPs stated that they now felt they had more access to spirometry than in the past.

### Methods for teaching about respiratory medicine

All groups mentioned the importance of bedside teaching and learning in a clinical context. Medical students specifically wanted positive encouragement and instruction from colleagues not necessarily the most senior but the most experienced.

"There are so many nurses, ambulance men and so on. Sometimes they have vastly more experience than some doctors in certain things and so you know, some of them might be better teachers at the end of the day, so they have more experience with certain tools and things then I am all for that" (UG-3)

Junior postgraduates cited the need for more practical training. Specialist registrars commented that more information about the prior teaching given to their junior colleagues and students would help them tailor their teaching more closely to individual learner needs. GPs wanted training focused to their specific needs. GPs also stated the usefulness of basic retraining.

## Discussion

Our focus group work has shown that final year medical undergraduates and junior hospital staff rarely have any systematic approach to the symptom of breathlessness or the differential diagnosis of lung disease. When asked about their approach, most responded with their feelings regarding the emergency situation where any structure reported is that of resuscitation rather than diagnosis. A potential failing of current teaching is that undergraduates and trainees anticipate that much of their future work is going to be involved in acute care and describe approaches relevant to emergency departments. In reality much of their professional work will be concerned with the care of patient (often elderly) with long-term illness. This is especially the case in respiratory medicine where the burden of chronic ill-health due to asthma, COPD, diffuse parenchymal lung disease (DPLD), bronchiectasis, and cystic fibrosis amongst others is considerable.

It is noteworthy that, even when directed to consider cases of chronic breathlessness presenting outside the emergency room, only SpRs with an existing interest in respiratory medicine and GPs expressed their awareness of the potential value of spirometry. When its value was mentioned to more junior trainees, they commented that they rarely saw it used and that their seniors did not appear to value its role. However, one reason for this may be the focus of students and juniors on emergency presentations, where spirometry would not have a key role to play in immediate patient management. In contrast, senior trainees did value spirometry as an important diagnostic tool, but their enthusiasm appears not to be systematically passed on to junior colleagues, possibly because it may be perceived as so fundamental and routine by senior staff that they fail to overtly stress its importance when teaching (see example comment in results section above). General practitioners taking part in the focus groups were highly aware of the value of spirometry and this may, of course, reflect recent inclusion of accurate diagnosis of COPD by use of spirometry as a quality marker in the UK National Health Service General Practitioners' contract. Interestingly, despite their awareness of, and enthusiasm for spirometry, the general practitioners in our focus group commented on the need for retraining, which would be commensurate with a study by Bolton *et al *which showed that only 33% of general practices were confident at interpreting spirometry and 58% were confident at using spirometers [21].

There are obvious differences between primary and secondary care. A study by Janson *et al *[22] showed that only 27% of physicians always used spirometry to diagnose asthma, in comparison to 73% of specialists, whilst 68% of primary care doctors used spirometry to monitor patients for asthma compared to 88% of specialists. However, as already discussed, non-respiratory specialists may also overlook or misdiagnose airways narrowing in the absence of spirometry [4].

At least one other paper has commented that spirometry is not taught to the same level as other diagnostic methods such as undertaking a physical exam or interpreting electrocardiograms [23]. Spirometry should be taught within the clinical context so that its value is apparent to trainees. All our participants commented on the importance of patient-centred teaching and its value. Whilst it has been shown that further education increases the use of spirometry by general practitioners [24] and also improves their capacity to diagnose clearcut pathologies [25], there is less literature on the teaching of spirometry to junior postgraduates and undergraduate medical students. New methods for teaching spirometry should be evaluated and we have previously shown that e-learning can have advantages in this context and is especially beneficial in helping trainees with data interpretation [26].

Perhaps the clearest message from this study, is that the perceived attitudes of educators and mentors are crucial drivers of the behaviours and attitudes of their junior trainees. There is a substantial literature on professionalism in which the attitudes and behaviours of tutors and consultants are frequently identified as elements of the "hidden curriculum" which can either reinforce, or undermine the objectives of the overt curriculum [27,28]. Similarly, the willingness of undergraduate students to engage with medical ethics has been shown to be increased if they have encountered positive role models during the their training [29]. However, what is unusual about the current study is that the potential role models (senior respiratory trainees and GPs) were extremely enthusiastic proponents of spirometry, but this enthusiasm was not perceived by their junior trainees. This may in part be explained by trainees also being exposed to role models who were not necessarily specialists in respiratory medicine. An in-depth exploration of the perceptions of spirometry amongst senior hospital doctors who are not respiratory specialists would be useful to establish whether this was the case. A further potential limitation of our study is that our GPs, who were uniformly enthusiastic about spirometry, may not have been typical of all in primary care, since they were GPs with a special interest in teaching undergraduates and were recruited whilst attending an educational update session. A third potential limitation was that we conducted only a single focus group with participants at each level of seniority and thus, our sample size was modest. Because we did not conduct multiple groups with each grade of doctor or trainee, it is possible that we did not achieve complete saturation of all themes. For example, in the theme of training, junior doctors expressed a *desire *for training whilst specialist registrars discussed *offering *training. It is possible that had we been able to conduct multiple focus groups with each level of seniority or mixed grades of doctor, further codes would have been identified. However, we choose not to mix groups, as we considered that the presence of more senior staff might well inhibit the junior ones from expressing their honest opinions.

Nevertheless, overall our results suggest that that respiratory physicians who find themselves in any supervisory or educational role should take every possible opportunity to explicitly discuss the value of spirometry with their junior colleagues and it is possible that the availability of good e-learning materials may also better induce confidence in interpretation [26].

## Conclusions

Both medical undergraduates and junior postgraduates require explicit instruction regarding the value of spirometry in the diagnosis and management of respiratory patients. They also need practical experience in using equipment of the type commonly found on wards and in GP surgeries and practice in interpreting the results. However, even comprehensive training is unlikely to be beneficial unless the senior staff who act as role models and trainers are observed by their trainees to use spirometry themselves.

## Competing interests

The authors declare that they have no competing interests.

## Authors' contributions

All authors contributed equally to the design and execution of this study. All authors read and approved the final manuscript.

## Pre-publication history

The pre-publication history for this paper can be accessed here:

http://www.biomedcentral.com/1471-2466/11/37/prepub

## References

[B1] Hamers R Bontemps S van den AM Souza R Penaforte J Chavannes N Chronic obstructive pulmonary disease in Brazilian primary care: Diagnostic competence and case-finding Prim Care Respir J 2006 15 5 299 306 10.1016/j.pcrj.2006.07.008 16978923 PMC6730828

[B2] Fauzi A Knowledge and practice of medical doctors on chronic obstructive pulmonary disease: a preliminary survey from a state hospital Med J Malaysia 2003 58 2 205 212 14569740

[B3] Alvarez Luque I Flor Escriche X Rodriguez Mas M Gallego Alvarez L Fraga Martinez M Sanchez Pinacho L Do we forget asthma as a chronic illness in our primary care consultations? Aten Primaria 2004 33 381 386 10.1157/13060756 15117633 PMC7668823

[B4] Zaas D Wise R Wiener C Airway Obstruction Is Common but Unsuspected in Patients Admitted to a General Medicine Service Chest 2004 125 1 106 111 10.1378/chest.125.1.106 14718428

[B5] Volkova NB Kodani A Hilario D Munyaradzi SM Peterson MW Spirometry utilization after hospitalization for patients with chronic obstructive pulmonary disease exacerbations Am J Med Qual 2009 24 1 61 66 19139465 10.1177/1062860608326417

[B6] Buffels J Degryse J Liistro G Diagnostic certainty, co-morbidity and medication in a primary care population with presumed airway obstruction: the DIDASCO2 study Prim Care Respir J 2009 18 1 34 40 18701969 10.3132/pcrj.2008.00047 PMC6619038

[B7] Moore PL Practice management and chronic obstructive pulmonary disease in primary care Am J Med 2007 120 8 Suppl 1 S23 S27 17678940 10.1016/j.amjmed.2007.04.009

[B8] Lange P Andersen KK Munch E Sorensen TB Dollerup J Kasso K Quality of COPD care in hospital outpatient clinics in Denmark: The KOLIBRI study Respir Med 2009 103 11 1657 1662 10.1016/j.rmed.2009.05.010 19520562

[B9] Barton C Proudfoot J Amoroso C Ramsay E Holton C Bubner T Management of asthma in Australian general practice: care is still not in line with clinical practice guidelines Prim Care Resp J 2009 18 2 100 105 10.3132/pcrj.2008.00059 PMC6619049 18830522

[B10] Weidinger P Nilsson JL Lindblad U Adherence to diagnostic guidelines and quality indicators in asthma and COPD in Swedish primary care Pharmacoepidemiol Drug Saf 2009 18 5 393 400 10.1002/pds.1734 19288473

[B11] Hueto J Cebollero P Pascal I Cascante JA Eguia VM Teruel F Spirometry in primary care in Navarre, Spain Arch Bronconeumol 2006 42 7 326 331 16945262 10.1016/s1579-2129(06)60541-7

[B12] Yawn BP Wollan PC Knowledge and attitudes of family physicians coming to COPD continuing medical education Int J Chron Obstruct Pulmon Dis 2008 3 2 311 317 18686740 10.2147/copd.s2486 PMC2629969

[B13] Lusuardi M Garuti G Massobrio M Spagnolatti L Bendinelli S Heart and lungs in COPD. Close friends in real life--separate in daily medical practice? Monaldi Arch Chest Dis 2008 69 1 11 7 18507194 10.4081/monaldi.2008.406

[B14] Fromer L Cooper CB A review of the GOLD guidelines for the diagnosis and treatment of patients with COPD Int J Clin Pract 2008 62 8 1219 1236 10.1111/j.1742-1241.2008.01807.x 18547365

[B15] Walters JA Hansen EC Johns DP Blizzard EL Walters EH Wood-Baker R A mixed methods study to compare models of spirometry delivery in primary care for patients at risk of COPD Thorax 2008 63 5 408 414 10.1136/thx.2007.082859 18024537

[B16] Wickstrom GC Kolar MM Keyserling TC Kelley DK Xie SX Bognar BA Confidence of Graduating Internal Medicine Residents to Perform Ambulatory Procedures Journal of General Internal Medicine 2000 15 6 361 365 10.1046/j.1525-1497.2000.04118.x 10886469 PMC1495472

[B17] Cranston JM Crockett AJ Moss JR Pegram RW Stocks NP Models of chronic disease management in primary care for patients with mild-to-moderate asthma or COPD: a narrative review Med J Aust 2008 188 8 Suppl S50 S52 18429736 10.5694/j.1326-5377.2008.tb01744.x

[B18] Barr RG Celli BR Martinez FJ Ries AL Rennard SI Reilly JJ Jr Physician and patient perceptions in COPD: the COPD Resource Network Needs Assessment Survey Am J Med 2005 118 12 1415 16378794 10.1016/j.amjmed.2005.07.059

[B19] Petty TL Benefits of and barriers to the widespread use of spirometry Curr Opin Pulm Med 2005 11 2 115 120 15699782 10.1097/01.mcp.0000150948.13034.78

[B20] Bryman A Qualitative data analysis, Chapter 22 in "Social Research Methods" 2008 Third Oxford University Press; Oxford

[B21] Bolton CE Ionescu AA Edwards PH Faulkner TA Edwards SM Shale DJ Attaining a correct diagnosis of COPD in general practice Respir Med 2005 99 4 493 500 10.1016/j.rmed.2004.09.015 15763457

[B22] Janson SL Fahy JV Covington JK Paul SM Gold WM Boushey HA Effects of individual self-management education on clinical, biological, and adherence outcomes in asthma Am J Med 2003 115 8 620 626 10.1016/j.amjmed.2003.07.008 14656614

[B23] Petty TL Harm From Spirometry? Chest 2006 130 5 1629 1630 17099055 10.1378/chest.130.5.1629

[B24] Kaminsky DA Marcy TW Bachand M Irvin CG Knowledge and use of office spirometry for the detection of chronic obstructive pulmonary disease by primary care physicians Respir Care 2005 50 12 1639 1648 16318645

[B25] Chavannes N Schermer T Akkermans R Jacobs JE van de GG Bollen R Impact of spirometry on GPs' diagnostic differentiation and decision-making Respir Med 2004 98 11 1124 1130 10.1016/j.rmed.2004.04.004 15526814

[B26] Smith SF Roberts NJ Partridge MR Comparison of a web-based package with tutor-based methods of teaching respiratory medicine: subjective and objective evaluations BMC medical education 2007 7 1 41 10.1186/1472-6920-7-41 17976233 PMC2180172

[B27] Goldie J Dowie A Cotton P Morrison J Teaching professionalism in the early years of a medical curriculum: a qualitative study Med Educ 2007 41 6 610 617 10.1111/j.1365-2923.2007.02772.x 17518842

[B28] Stephenson AE Adshead LE Higgs RH The teaching of professional attitudes within UK medical schools: Reported difficulties and good practice Med Educ 2006 40 11 1072 1080 10.1111/j.1365-2929.2006.02607.x 17054616

[B29] Lynoe N Lofmark R Thulesius HO Teaching medical ethics: what is the impact of role models? Some experiences from Swedish medical schools J Med Ethics 2008 34 4 315 316 10.1136/jme.2007.021147 18375688

